# Women, Gender and Mental Health: What factors help mentally ill birth mothers navigate Child Welfare Services during the perinatal period?

**DOI:** 10.1192/j.eurpsy.2025.2437

**Published:** 2025-08-26

**Authors:** H. L. A. C. R. Wanigasekera, A. Buist

**Affiliations:** 1 Psychiatry, Austin Health; 2 Psychiatry, University of Melbourne, Melbourne, Australia

## Abstract

**Introduction:**

**Women, Gender and Mental Health: What factors help mentally ill birth mothers navigate Child Welfare Services during the perinatal period?**

**Chamali Wanigasekera** ^**1**^ MBBS, AMC, MPsych, FRANZCP

**Anne Buist** ^**1,2**^ MBBS, MMed, MD, FRANZCP

1. Austin Health, Heidelberg, Victoria, Australia
2. University of Melbourne, Victoria, Australia

Email: Chamali.WANIGASEKERA@austin.org.au; a.buist@unimelb.edu.au

**Background:**

Mental Illness is common in the perinatal period. Given the dependency of infants, additional factors such as lack of support, family violence and comorbid drug use may place the infant at significant physical and psychological harm. Anecdotal information suggests that many of these women do not receive adequate support during the perinatal period. However, the factors that predict and protect families, enabling them to stay together and function in a “good enough “manner, remain unclear.

**Objectives:**

**Aims:** To review the current literature examining protective service involvement in the women who present with mental illness in the perinatal period.

**Methods:**

A systematized review of the literature was conducted through a comprehensive search of databases for psychosocial and medical research (MEDLINE, PsycINFO, Embase, Emcare, Cochrane Library) and a targeted search of the grey literature to select the relevant studies that meet the inclusion criteria. Original papers were included if they were written in English and published before September 1, 2022. Sixteen studies were selected for inclusion.

**Results:**

Protective and predictive factors that help mentally ill mothers to continue as primary caregivers will be presented. The factors that determine risks for child protective involvement and child removal in mothers with mental illness are also discussed.

**Conclusions:**

The results of this research will provide recommendations on how to deliver sensitive perinatal mental health care for mothers already involved with the Child Welfare Services, aiming to minimize child removal.

**Disclosure of Interest:**

None Declared

